# The Highs and Lows of an Unknown Pheochromocytoma in an Elderly Patient

**DOI:** 10.1155/2019/5707968

**Published:** 2019-07-07

**Authors:** Christina N. DiMaria, Lorena I. Rasquin, Wikien A. Hung Pinto

**Affiliations:** Department of Internal Medicine, Einstein Medical Center, Philadelphia, PA, 19141, USA

## Abstract

Pheochromocytomas are rare catecholamine producing neuroendocrine tumors. The incidence of these tumors is estimated to affect 0.8 per 100,000 person-years and is most common in the fourth to fifth decade of life with equal prevalence in men and women. We describe a case of an 84-year-old male who presented with cycling episodes of severe hypertension and hypotension after an elective cardiac catheterization. Workup of the labile blood pressure revealed a large suprarenal mass and free serum metanephrines (MN) 104 nmol/L (reference range 0.0-0.49 nmol/L) and normetanephrines (NMN) of 24 nmol/L (reference range 0.0-0.89 nmol/L), confirming the diagnosis of a pheochromocytoma. The patient's labile blood pressure was a challenge to manage medically and improved only after aggressive hydration and an alpha-adrenergic antagonist. Of note, this is the second eldest patient known to be published to date with a pheochromocytoma.

## 1. Introduction

Pheochromocytomas are rare catecholamine producing neuroendocrine tumors. The incidence of these tumors is estimated to affect 0.8 per 100,000 person-years and are most common in the fourth to fifth decade of life with equal prevalence in men and women [1, 2]. Most of these tumors (85%) arise from the adrenal medulla. Those arising from the extra-adrenal neural ganglia are called paragangliomas.

Due to a large production of catecholamines, such as epinephrine, norepinephrine, and dopamine, patients can present with the classic triad of symptoms of sweating, headaches, and tachycardia [3]. Other patients can present during a pheochromocytoma induced “spell,” with sudden onset of paroxysmal hypertension that is self-limited [4]. However, it is important to note most patients do not present with the classic triad or during a pheochromocytoma spell [5, 6]. The diagnosis of pheochromocytoma is made by biochemical testing in urine and serum for metanephrine (MN) and normetanephrine (NMN) end products of catecholamine metabolism, followed by imaging studies such as a computerized tomography (CT) scan.

In this report, we describe a case of an 84-year-old male diagnosed with a pheochromocytoma and cycling episodes of hypertension and hypotension. Of note, this is the second eldest patient known to be published to date with a pheochromocytoma.

## 2. Case Presentation

We present the case of an 84-year-old male with past medical history of hypertension, benign prostatic hyperplasia, former alcohol and cocaine abuse, chronic kidney disease stage 3A, severe malnutrition, and frequent falls who presented from a nursing home for elective left heart catheterization. The patient had an NSTEMI two months earlier but had refused catheterization at the time with new left ventricular systolic dysfunction and an ejection fraction of 45%.

During the catheterization, the patient developed hypertensive emergency with flash pulmonary edema and the procedure had to be aborted. He was admitted to the Cardiac Critical Care Unit for hypertensive emergency and was started on antihypertensive drips. Urine drug screen was negative. He was weaned off the drips after 36 hours. After which he developed labile blood pressures, fluctuating in minutes, and ranging from 68/31 mmHg to 323/115 mmHg with his heart rate ranging from 90 bpm to 140 bpm. These blood pressure readings were measured in four extremities with a manual manometer and confirmed with an arterial line. During the rapid shifts in blood pressure the patient would develop blank staring episodes and nonresponsiveness that would last approximately 30 seconds to 1 minute. An immediate head CT showed no acute ischemic changes, hemorrhagic stroke, or cerebral edema. Electroencephalogram showed generalized slowing consistent with moderate metabolic encephalopathy. In addition, the patient developed acute kidney injury with maximum creatinine of 2.6 mg/dl from a baseline of 1.3 mg/dl.

Workup of the labile blood pressure, including an abdominal CT scan without contrast because of the patient's kidney injury revealed a large 9.4 x 8.7 x 8.1 cm round, slightly lobulated left suprarenal mass with mixed cystic and solid components and no calcifications with Hounsfield units ranging from 15 to 45. Free serum metanephrines (MN) were 104 nmol/L (reference range 0.0-0.49 nmol/L) and normetanephrines (NMN) were 24 nmol/L (reference range 0.0-0.89 nmol/L), with a ratio of MN to NMN of 4:1, which were drawn at the peak of a hypertensive cycle. Because of the labile blood pressures, the patient was on no antihypertensive medications, including alpha or beta blockers or calcium channel antagonists, at the time when then the serum and urine metanephrines were drawn. His UDS was negative for cocaine and the patient was not on tricyclic antidepressant or other drugs that can cause false positive elevations of plasma and urinary catecholamines or metanephrines [7]. The findings confirmed the diagnosis of pheochromocytoma.

The patient was started on prazosin and, however, developed worsening hypotension again, even while receiving aggressive fluid resuscitation. After the patient was euvolemic, the prazosin was able to be titrated up to goal with stabilization of his blood pressure. Due to his advanced age, severe malnutrition, no family support, worsening mental status, and comorbidities he was deemed not an appropriate surgical candidate. Patient was discharged under hospice care.

## 3. Discussion

In this case report we present the second eldest patient to date, based on literature review, to be diagnosed with a pheochromocytoma at age 84 years old. The eldest patient to date was an 87-year-old male from Buffet et al.'s study on germline mutations in a metastatic paragangliomas [8]. Previously, Chenguan et al. presented a case of a giant malignant pheochromocytoma in an 81-year-old male that measured 13.5x10.6x9.8 cm [9]. Our patient's pheochromocytoma was comparable in size and there was suspect it had been partially controlled and disguising as primary hypertension, as the patient was on prazosin for benign prostatic hyperplasia and on multiple antihypertensive medications.

In our patient's case, he did not receive perioperative blockade as his pheochromocytoma was unknown at the time of the procedure, his prazosin was held, and the stress of the cardiac catheterization precipitated a pheochromocytoma spell, causing a hypertensive emergency. He was initially started on intravenous nitroglycerin that was changed to nicardipine for better blood pressure control. Per the Endocrine Clinical Practice Guidelines, patients with functional pheochromocytomas and paragangliomas should receive perioperative blockade in addition to a high-sodium diet and fluid intake prior to the surgery [10]. Perioperative blockade consists of alpha-adrenergic receptor blockers as the first-choice drug class to minimize perioperative complications and calcium channel blockers are the most often used add on drug to control blood pressure. Beta-adrenergic receptor blockers are indicated to control tachycardia, but only after alpha-adrenergic receptor blockade has been achieved.

By day two the patient's blood pressure began to wildly fluctuate. The intravenous nicardipine was discontinued. From the standpoint of the medical team, this labile blood pressure was extremely challenging to manage. Each time the team tried to intervene on the elevated blood pressure his blood pressure suddenly plummeted and vice versa. There was concern which extreme was more harmful to the patient and was there a balance in treatment.

There are multiple cases describing such cyclic episodes of hypotension and hypertension with pheochromocytomas [11–13]. A review by Kobal et al. of seven cases found an association of labile blood pressures with tumors that predominantly secrete epinephrine [14]. This corresponds with our case's findings, showing serum catecholamine markers as primarily metanephrine, a metabolite of epinephrine.

There are a few proposed mechanisms for this cyclic blood pressure; however the exact mechanism is uncertain. One proposed mechanism is based on autoregulation by baroreceptors. A surge in catecholamines, primarily epinephrine, would cause vasoconstriction and decreased blood volume leading to decreased cardiac output triggering further release of catecholamines. The baroreceptors would then sense the vasoconstriction causing negative feedback of the sympathetic and parasympathetic nervous system resulting in hypotension [15]. Another proposed mechanism is that posture affects tumor secretions. Oishi et al. proposed that an increase in intra‐abdominal pressure leads to a surge in catecholamines release and noted that when patients lay supine that their blood pressure would fluctuate [10]. While other theories suggest intravascular volume depletion and ischemic response of the central nervous system as cause of the varying blood pressures [16], our patient's blood pressure remained labile after starting prazosin and only stabilized after aggressive fluid resuscitating, supporting the latter mechanism.

There was discussion which alpha-blockade to initiate. The patient was on prazosin, a short acting alpha-1- adrenergic blocker, for BPH with blood pressure control prior to admission. Phenoxybenzamine, an irreversible, long acting, and nonselective alpha receptor antagonist, is the preferred drug for preoperative preparation to control blood pressure. Doxazosin, a selective alpha-1-adrenergic blocker, was another option [10]. However, with the labile pressures and concern for hypotension, there was concern for using a long acting antagonist and prazosin was initiated because of its ability to be up titrated. In the end, the patient's blood pressure was able to be managed with fluid resuscitation and prazosin without the need for a second antihypertensive agent.

The only curative modality for pheochromocytoma is tumor excision, with a laparoscopic approach being the procedure of choice for patients with a solitary intra-adrenal pheochromocytomas without malignant radiologic features [10]. As noted, the patient's advanced age, severe malnutrition, worsening mental status, being with no family made him a poor surgical candidate. These factors also contributed to the decision to not proceed with genetic evaluation, as the Endocrine Clinical Practice Guidelines recommend all patients be engaged in shared decision-making for genetic testing [10].

## 4. Conclusion

In conclusion, pheochromocytoma can be seen in elderly patients. It is important to note that alpha-adrenergic antagonist medications may mask an insidious pheochromocytoma unless provoked by a stressor, like a surgery. The composition of the tumor secretions, epinephrine versus norepinephrine versus dopamine, can lead to varying presentations of patients that should trigger pheochromocytoma workup. Lastly, patients with pheochromocytomas that present with rapid cyclic fluctuations of hypertension and hypotension may benefit from fluid repletion in addition to an alpha-adrenergic antagonist.

## Data Availability

The case report findings, including case demographics, imaging, vital signs, and lab results, used to support the findings of this report are available from the corresponding author upon request.
